# Synthesis, Spectroscopic, Molecular Structure, and Antibacterial Studies of Dibutyltin(IV) Schiff Base Complexes Derived from Phenylalanine, Isoleucine, and Glycine

**DOI:** 10.1155/2014/716578

**Published:** 2014-11-27

**Authors:** Har Lal Singh, Jangbhadur Singh

**Affiliations:** Department of Chemistry, Faculty of Engineering & Technology, Mody University of Science and Technology, Laxmangarh, Sikar, Rajasthan 332311, India

## Abstract

New series of organotin(IV) complexes and Schiff bases derived from amino acids have been designed and synthesized from condensation of *1H*-indole-2,3-dione, 5-chloro-*1H*-indole-2,3-dione, and *α*-amino acids (phenylalanine, isoleucine, and glycine). All compounds are characterized by elemental analyses, molar conductance measurements, and molecular weight determinations. Bonding of these complexes is discussed in terms of their UV-visible, infrared, and nuclear magnetic resonance (^1^H, ^13^C, and ^119^Sn NMR) spectral studies. The results suggest that Schiff bases behave as monobasic bidentate ligands and coordinate with dibutyltin(IV) in octahedral geometry according to the general formula [Bu_2_Sn(L)_2_]. Elemental analyses and NMR spectral data of the ligands with their dibutyltin(IV) complexes agree with their proposed distorted octahedral structures. Few representative compounds are tested for their in vitro antibacterial activity against Gram-positive (*B. cereus*, *Staphylococcus* spp.) and Gram-negative (*E. coli*, *Klebsiella* spp.) bacteria. The results show that the dibutyltin complexes are more reactive with respect to their corresponding Schiff base ligands.

## 1. Introduction

Amino acids and their compounds with different metal ions play an important role in pharmaceutical industries [1–5]. A number of studies have indicated that biologically active compounds become more bacteriostatic and carcinostatic upon chelation. Some drugs show increased activity when administered as metal chelates and inhibit the growth of tumor. Moreover, the development in the field of bioinorganic chemistry has increased the interest in Schiff base complexes, since it has been recognized that many of these may serve as models for biologically important species [6–9]. During the past few decades, research about new organotin compounds has increased dramatically, most likely due to their diverse biological applications [10–13]. A huge interest in metal complexes of Schiff bases derived from amino acids and salicylaldehyde has emerged due to their structural, magnetic, and electrochemical properties, as well as their potential use as models for a number of important biological systems [14–16]. Among their several biological functions, they show antimicrobial, antimalarial, antiproliferative, chemotherapeutic, radiopharmaceutical, insulin-mimetic, and fungicidal properties [17–23]. One of the most important bioinorganic chemistry research areas in organotin compounds is the investigation of their cytotoxic and antitumor activities [24]. Moreover, tin(IV) complexes characterized by the presence of one or more carbon-tin bonds have proved to be cytotoxic against the breast adenocarcinoma tumor and the colon carcinoma [25]. In general, the toxicity of organotin compounds seems to increase with the chain length of the organic alkyl groups, which are often more active than aryl ones [26, 27].

Although the major uses of these derivatives are well known for their versatile and significant important biological and pharmaceutical activities and as wood preservatives and pesticides [28–34], the organotin(IV) compounds possess potential applications in the field of industrial and medicinal chemistry. Organotin(IV) complexes have been extensively studied due to their coordination geometries as well as structural diversity. In view of this, the synthesis of organotin(IV) complexes of Schiff bases derived from the condensation of* 1H*-indole-2,3-dione and 5-chloro-*1H*-indole-2,3-dione with different amino acids (phenylalanine, isoleucine, and glycine) derivatives is reported herein. The characterization of the complexes was realized by elemental analysis and spectroscopic (UV, IR, ^1^H, ^13^C, and ^119^Sn NMR) studies. Their antibacterial activities were screened against various Gram-positive and Gram-negative bacteria.

## 2. Experimental

All reagents were commercially available (Aldrich or Merck) and used as supplied. Solvents were purified and dried according to standard procedures and moisture was excluded from the glass apparatus using CaCl_2_ drying tubes. Melting points were determined in open glass capillaries and were uncorrected. The ligands were prepared by the condensation of isatin and 5-chloroisatin with amino acids (phenylalanine, isoleucine, and glycine) as described earlier [35].

### 2.1. Analytical Methods and Spectral Measurements

Tin was estimated gravimetrically as SnO_2_. CHN analyses were carried out on a Perkin Elmer 2400 Elemental Analyser. Molecular weight determinations were carried out by the Rast camphor method. Molar conductance measurements were made in anhydrous dimethylformamide at 25 ± 5°C using a Systronics conductivity bridge model 305. The electronic spectra were recorded in DMSO on a Thermo UV1 spectrophotometer. IR spectra were recorded on a Perkin Elmer Spectrum SP-2 Fourier transform spectrophotometer using KBr pellets (4000–400 cm^−1^). ^1^H and ^13^C NMR spectra were recorded on a Bruker Avance-II (400 MHz) FTNMR spectrometer using DMSO-d_6_ as solvent at 400 MHz and 100 MHz, respectively. TMS was used as internal reference for ^1^H NMR and ^13^C NMR. The ^119^Sn NMR spectra with proton noise decoupling were recorded on a Bruker Avance-II spectrometer using dry DMSO as the solvent at 149.21 MHz and tetramethyltin (TMT) as an external standard. X-ray powder patterns were obtained with a SIEMENS D-5000 X-ray diffractometer using Cu K*α* radiation (*l* = 1.5405984 Å) which was operated at 30 kV and 15 mA.

### 2.2. 3D Molecular Modeling Analysis

The molecular modeling of a representative compound was carried out on a CS Chem3D Ultra Molecular Modeling and using MM2 analysis program [36]. It is an interactive graphics program that allows rapid structure building, geometry optimization with minimum energy, and molecular display. Correct sequence of atoms was obtained to get reasonable low energy molecular models to determine their molecular representation in three dimensions. Complications of molecular transformations could be explored using output obtained. An attempt to gain a better insight into the molecular structure of compounds, geometric optimization, and conformational analysis was performed using MM2 force field [37].

### 2.3. Syntheses of Organotin(IV) Complexes

Dibutyltin(IV) oxide (0.280 g; 1.125 mmol) was added to the calculated amount of the ligands (0.491−0.829 g; 2.250 mmol) in a 1 : 2 molar ratio in dry benzene (75 mL), with methanol (25 mL) mixture as reaction medium. The contents were refluxed on a fractionating column for about 5–7 hours. The water liberated in the reaction was removed azeotropically with benzene. On completion of the reaction, the resulting products were rendered free from solvent and then washed repeatedly with dry cyclohexane. The crystalline solids were separated out and purified by recrystallization from the same solvent. The products so formed were finally dried in vacuum at 40 ± 5°C for 2-3 hours. The purity of the complexes was checked by TLC using silica gel type G as adsorbent. Their physical properties and analytical data were recorded in Table 1.

### 2.4. Antibacterial Activity

Synthesized compounds were screened for their antibacterial activity against Gram-positive (*B. cereus*,* Staphylococcus *spp.) and Gram-negative (*E. coli*,* Klebsiella *spp.) bacteria at two concentrations 500 ppm and 200 ppm. The compounds were dissolved in DMSO. The in vitro antibacterial activity of the free ligands and their tin complexes was tested using the agar well diffusion method [38]. Using a sterile cork borer (5 mm in diameter) wells were made in each agar plate, more than 0.1 mL of the tested compounds were poured into three wells, and the dishes were incubated at 28 ± 2°C for 24 hours. The growth of the microorganisms was inhibited by diffusion of the test solutions and then the inhibition zone around the well was measured. The antibacterial activity of each compound was compared with standard antibiotics such as streptomycin. DMSO was used as a control under the same conditions to test organisms and no activity was found.

## 3. Results and Discussion

**Scheme 1:**
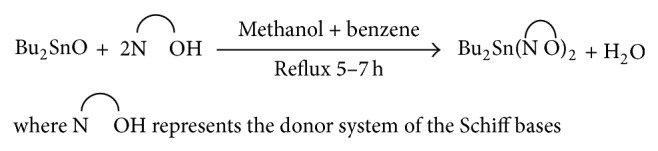
Representative equation illustrating the formation of dibutyltin(IV) complexes.

New organotin(IV) complexes were synthesized by the reactions of dibutyltin(IV) oxide with Schiff bases, which have been carried out in 1 : 2 molar ratios using anhydrous benzene and absolute methanol in a 3 : 1 ratio as solvent. These reactions proceed with the liberation of water, which was azeotropically removed. The results of elemental analysis (C, H, and N) with molecular formula and the melting points are presented in Table 1. The results obtained are in good agreement with those calculated for the suggested formula. The scheme of the organotin(IV) complexes preparation is given below (Scheme 1).

The above reactions were found to be quite facile and could be completed in 5–7 h of refluxing. All these complexes are intensively coloured and are solids. They are soluble in methanol, DMF, and DMSO. The compounds were dissolved in DMF and molar conductance 10^−3 ^M of solution at 25°C was measured. The molar conductance values of the complexes fall in the range from 10.32 to 20.14 Ω^−1^ cm^2 ^mol^−1^ indicating that these compounds are nonelectrolytes.

### 3.1. Electronic Spectra

The spectra of the ligands and their complexes were recorded in dry DMSO. The various bands observed were assigned to interligand and charge transfer of *n*–*π* ^*^ transitions according to their energies and intensities. Two intense maxima are observed in the complexes at 210–232 and 375–382 nm ranges which may be assigned to *n*–*π* ^*^ transition of the carboxylate [39] and of the C=N chromophore. The appreciable shifting observed in the *n*–*π* ^*^ transition (~370 nm) is due to the polarization in the C=N bond caused by tin-ligand electron interaction. This clearly indicates the coordination of the azomethine nitrogen with the tin atom. A band in the region 355–338 nm in the spectra of the Schiff bases and complexes is likely to be the secondary band of the benzene ring coupled with the intramolecular charge transfer transition taking place within the ligand moiety. Furthermore, sharp bands were observed in the region 246–332 nm in the spectra of the complexes which could be assigned to the charge transfer transition from ligand to tin atom [40].

### 3.2. Infrared Spectra

The IR spectra of these compounds have been recorded in the range of 4000–400 cm^−1^. Tentative assignments have been made on the basis of earlier publications and the important data are listed in Table 2. The absorptions of interest in the spectra of the complexes are *ν*(COO), *ν*(Sn–C), and *ν*(Sn–O). The position and the intensities of these peaks are expected to be changed on chelation. New peaks and quasi-peaks are also a guide to chelation. The IR spectra of all the ligands show the absence of bands at 3450 and 1750 cm^−1^ due to *ν*(NH_2_) group of amino acids and *ν*(C=O) of isatin. Instead, a new prominent band at 1635 ± 5 cm^−1^ due to azomethine *ν*(C=N) linkage appeared in all the ligands [12, 34], indicating that condensation between ketone moiety of isatin and that of amino group of amino acids has taken place resulting in the formation of the desired ligands (L^1^H−L^6^H). Moreover, comparison of the IR spectra of the ligands with their organotin(IV) complexes showed a major shift to lower wave numbers by 10−15 cm^−1^ in azomethine *ν*(C=N) at 1620 ± 5 cm^−1^ suggesting the involvement of the azomethine nitrogen with the organotin(IV) ion [9, 11, 32]. The appearance of a new band of medium intensity in the region ~552–540 cm^−1^ in all of the derivatives studied, which may be assigned to *ν*(Sn←N), further confirms the coordination of the amino nitrogen with the tin moiety.

The absence of the *ν*(COOH) in all the organotin(IV) compounds in the 2750–3100 cm^−1^ region and the presence of *ν*(Sn–O) in the 432–420 cm^−1^ range indicate deprotonation of the carboxylic acid group and consequent coordination of the carboxylate group with the tin metal as expected. IR stretching frequencies *ν*_as_(OCO) and *ν*_s_(OCO) for carboxylates have been utilized as a characteristic tool to confirm the mode of coordination through carboxylate oxygen and also to identify the nature (monodentate, bidentate, or bridging/chelating) of bonding of the carboxylate group. Unless the carboxylate group is branched at the *α*-carbon or the organic substituents at the tin atom are bulky, carboxylate groups in organotin(IV) carboxylates generally adopt a bridge structure in the solid state [41]. The IR stretching frequencies of the carboxylate groups are very important for determining their structures; namely, when there are interactions between the carbonyl oxygen atoms of the carboxylate groups and the tin atom, the asymmetric absorption vibration frequencies *ν*_as_(OCO) of the carboxylate groups decrease and the symmetric absorption frequencies *ν*_s_(OCO) increase. In the IR spectra of the title compounds, the carboxylate bands are observed in the characteristic regions for *ν*_as_(OCO) between 1610 and 1590 cm^−1^ and for *ν*_s_(OCO) between 1340 and 1325 cm^−1^ (Table 2). In the diorganotin(IV) complexes, the differences [*ν*Δ(OCO)] between *ν*_as_(OCO) and *ν*_s_(OCO) are more than 200 cm^−1^ indicating the covalent nature of the metal-oxygen bond. Ionic bonding and also bridging or chelation, therefore, can be excluded, and unidentate coordination of the carboxylic groups bonding to the metal must therefore be assumed. Thus, for diorganotin compounds the *ν*Δ(OCO) values were found to be more than 200 cm^−1^, which indicates that the carboxylate groups are chelated and bonded to the metal in a unidentate manner. Moreover, for complexes Δ*ν* below 200 cm^−1^ would be expected for bridging or chelating carboxylate, but greater than 200 cm^−1^ for the monodentate bonding carboxylate anions. Further evidence for the coordination with tin via oxygen atom was revealed by the presence of the *ν*(Sn–O) [33] stretching bands in the spectra of complexes in the region of 432–420 cm^−1^. Besides this, several new bands in the complexes observed at ~630 cm^−1^, ~540 cm^−1^, and ~430 cm^−1^ may be *ν*(Sn–C) [34], *ν*(Sn←N) [12], and *ν*(Sn–O) [38], respectively. The vacant 5d orbital of tin atoms tends toward high coordination with ligands containing lone pairs of electrons. The intensity observed for the symmetric Sn–CH_2_ stretching vibration at ~1440 cm^−1^ indicates a bent C–Sn–C moiety for the dibutyltin complexes. These data are consistent with a distorted octahedral configuration for the tin atom.

### 3.3. ^1^H NMR Spectra

The chemical shifts (*δ* ppm) of various protons in the complexes in DMSO-d_6_ solution are given in Table 3. The conclusions drawn from ^1^H NMR spectral studies lend further support to the mode of bonding discussed above. The absence of a signal at *δ* 12.00−11.00 ppm due to the C(O)OH proton suggests the deprotonation of the carboxylic oxygen atom of the ligands on complexation. The ligands give a complex multiplet signal in the region *δ* 7.10−7.85 ppm for the aromatic protons and these remain almost at the same position in the spectra of the metal complexes. The appearance of signals due to NH protons at the same positions in the ligand and its complexes shows the noninvolvement of this group in coordination. The butyl protons attached to tin appear as multiplets due to –CH_2_–CH_2_–CH_2_– protons in the *δ* 1.65−1.15 ppm region and as a clear triplet or broad signal due to terminal methyl protons in the *δ* 0.80–0.85 ppm region with ^*3*^ *J*_*HH*_ = 7.35 Hz. The >CH− protons in the complexes of the ligands give signals in the *δ* 4.38−3.99 ppm region. The most important piece of information, obtained from ^1^H NMR values in these compounds, demonstrates that diorganotin(IV) complexes show the coordination number greater than four, probably six, in noncoordinating solvent. The number of protons of various groups calculated from the integration curves and the number of those calculated for the expected molecular formula agree with each other.

### 3.4. ^13^C NMR Spectra

The ^13^C NMR spectral data along with assignment of characteristic peaks of ligands and its diorganotin(IV) complexes are presented in Table 4. The signals due to the carbon atoms attached to the carboxylate oxygen and the azomethine groups in the ligands appear at ~*δ* 176 ppm and ~*δ* 163 ppm, respectively. However, in the spectra of the corresponding tin complexes, these appear at ~*δ* 185 ppm (carboxylate group) and at ~*δ* 155 ppm (azomethine group), respectively. The considerable shifts in the resonances of the carbon atoms attached to oxygen and nitrogen indicate the involvement of oxygen and nitrogen atoms in coordination. The alkyl carbons of Schiff base and organotin(IV) complexes appear well within the expected ranges and their assignments are given in Table 4. The heteronuclear coupling constant ^*1*^ *J*(^13^C–^119^Sn) in the studied organotin(IV) complexes is determined, which provides vital information about the coordination environment and the geometry of the complexes. In all the complexes, the alkyl group are observed with coupling constant of the values ^*1*^ *J*(^13^C–^119^Sn) are in the range 690.56–759.86 Hz. These values lie in a typical range for six-coordinated organotin(IV) complexes [42].

### 3.5. ^119^Sn NMR Spectra

**Figure 1:**
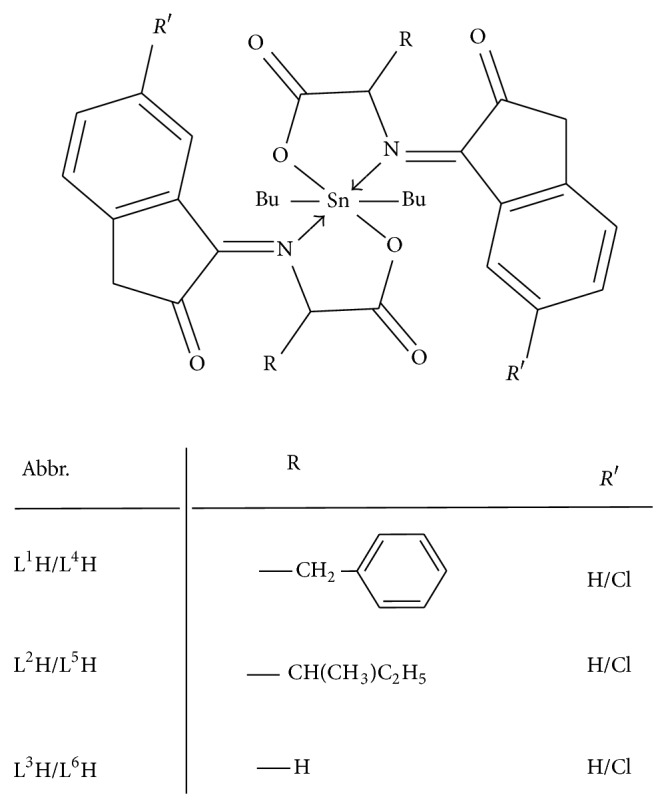
Structure of diorganotin(IV) complexes.

The ^119^Sn NMR chemical shifts are very sensitive to coordination number of tin and are generally shifted upfield on bonding to Lewis base. It has been reported [43, 44] that the ^119^Sn chemical shifts in the ranges from 200 to −60, from −90 to −190, and from −210 to −400 ppm are associated with four-, five-, and six-coordinated alkyltin(IV) compounds, and these ^119^Sn shifts are higher with aryltin(IV) compounds. In the ^119^Sn NMR spectra of Bu_2_Sn(L^1^)_2_, Bu_2_Sn(L^2^)_2_, and Bu_2_Sn(L^3^)_2_ recorded in DMSO-d_6_, the ^119^Sn chemical shifts are observed at *δ*  −265.4, −270.8, and −266.1 ppm, respectively, lying in the range of six-coordinated tin. The ^*1*^ *J *(^119^Sn–^13^C) values allow the calculation of the bond angle for the C–Sn–C fragment, which is in the order of ~117°, suggesting that the tin atom has a slightly distorted octahedral geometry (Figure 1); this behavior is in agreement with the values reported for hexacoordinated tin compounds [45].

### 3.6. X-Ray Powder Diffraction Studies

The X-ray diffraction studies have been performed on Philips PW 1710 automated X-ray powdered diffractometer. The experimental conditions employed in reading the pattern were as follows: the operating target voltage was 30 kV; the tube current was 15 mA. The X-ray from copper target was filtered with nickel, and monochromatic K*α* line of wavelength 1.540598 Å was obtained. Filtration reduces noise due to white radiation and increases resolution also.

**Figure 2:**
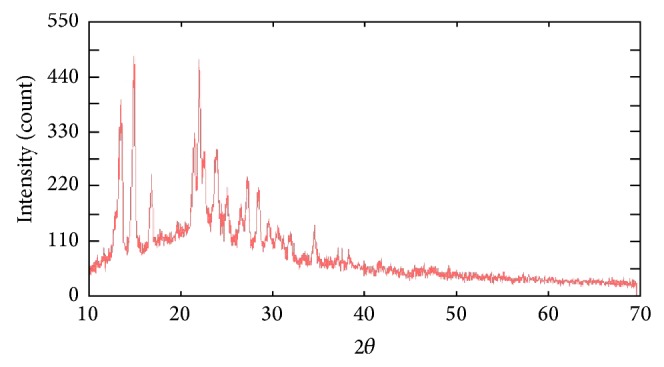
X-ray powder diffraction patterns of the compound [L^1^H].

**Figure 3:**
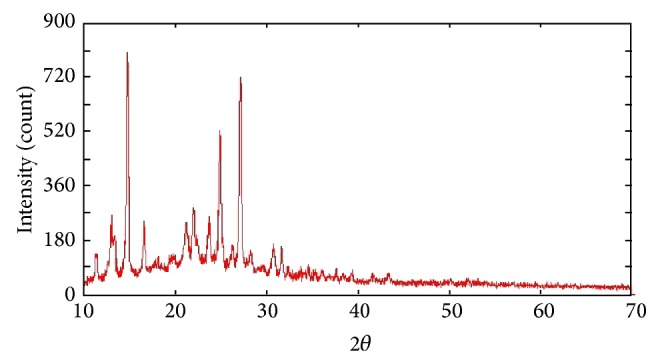
X-ray powder diffraction patterns of the compound [L^2^H].

The X-ray diffraction of L^1^H, L^2^H, L^3^H, and their metal complexes indicates the crystalline nature of the L^1^H, L^2^H, and L^3^H; Bu_2_Sn(L^3^)_2_ is shown to have amorphous nature. All the reflection has been indexed for *D* (particle size) using Scherrer's equation. Average crystallite size values of 22.484 nm and 32.439 nm were shown for L^1^H and L^2^H, respectively. These values of particle size and 2*θ* for each peak have been calculated with the help of the cell parameters and corresponding FWHM values. The lattice spacing, FWHM, and particle size for L^1^H and L^2^H have been found out and are given in Table 5. The diffractogram of compounds has been shown in Figures 2 and 3.

### 3.7. Molecular Structure

Molecular modeling helped to demonstrate the significant features, that is, molecular geometries, bond energy, and torsion angles of organometallic frameworks theoretically. Bond lengths, bond angles, and atomic coordinates depend on the hybridization of an atom and mode of bonding. Thus, molecular modeling was the blue print of three dimensional arrangements of atoms. Beside this, if deviations in bond distance, bond angles, or torsion angles are evidenced, specific electronic interactions can be detected and confirmed to the earlier spectral lines of evidence [46]. The bonding capabilities of atoms have impact on bond lengths and bond angles of concerned functional groups. Therefore, molecular models of complexes were demonstrated. Physical dimension of the molecules helped out to demonstrate the changes occurring during their topological assemblies. Energy minimization is used to find the structure with lowest energy using molecular mechanics analysis program [47, 48]; data analysis of bond lengths and bond angles is presented in Tables 6 and 7.

**Figure 4:**
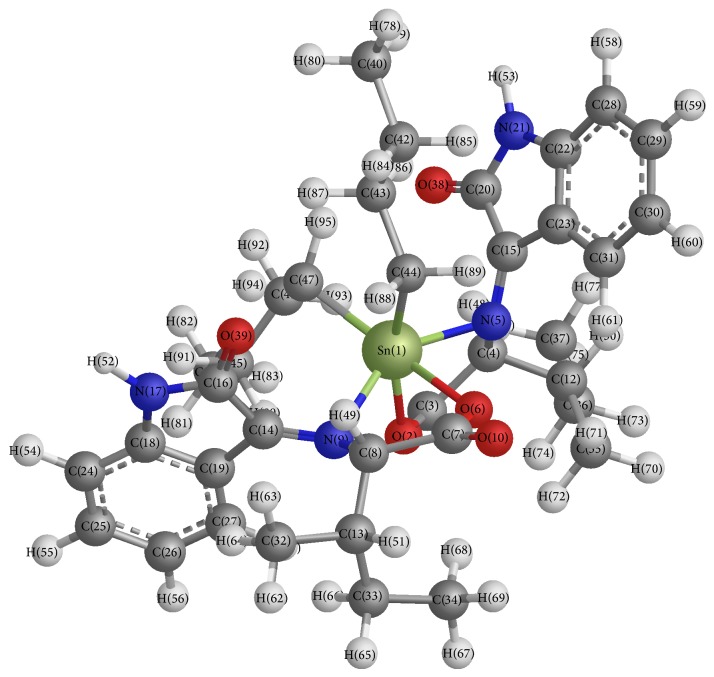
3D molecular structure of Bu_2_Sn(L^2^)_2_.

The molecular structure is shown in Figure 4. The deprotonated ligand is coordinated as bidentate ligand* via* the carboxylate oxygen and azomethine nitrogen atoms. The six coordination number is completed by two carbon atoms of butyl groups. The organic molecule acts as an anionic bidentate with the ON donors placed in the same side. Since the synthesized compounds are related and differ only in substituted R group, one compound Bu_2_Sn(L^2^)_2_ was theoretically studied. Based upon spectroscopic data, Sn(IV) compounds with O_2_N_2_ ligands generally adopt distorted octahedral geometry. The optimized structure for the compound Bu_2_Sn(L^2^)_2_ is shown in Figure 4 and pertinent bond parameters are given in Tables 6 and 7. The bond angles around tin atom, for example, C(47)–Sn(1)–C(44) angle of 117.15°, N(5)–Sn(1)–N(9) angle of 148.95°, and O(6)–Sn(1)–O(2) angle of 112.00°, in Bu_2_Sn(L^2^)_2_ are the representative of the distorted octahedral structure. The two Sn–O bond distances are close to being identical values. The calculated Sn–O bond distances of 2.0581/2.0644 Å in Bu_2_Sn(L^2^)_2_ are also close to the already reported Sn–O distances in {CH_2_N(Me)CH(Me)CH(Ph)O}_2_Sn (2.048/2.078) [49]. The two different Sn–N distances of Sn1–N5 and Sn1–N9 in compounds Bu_2_Sn(L^2^)_2_ are 2.1010/2.1050 Å, which are similar to the already reported structures, SalenH_2_Sn 2.0535(9) and 2.0369(8) Å) [50].

### 3.8. In Vitro Antibacterial Activity

Antibacterial screening of the synthesized compounds was carried out using four bacterial strains of Gram-positive (*B. cereus*,* Staphylococcus *spp.) and Gram-negative (*E. coli*,* Klebsiella *spp.) bacteria. The zones of inhibition values in Table 8 represent the mean value of the three readings with standard deviation. Streptomycin was used as a reference compound for antibacterial activities. These bacterial strains are used because they are known as common pathogens of human beings. The antimicrobial studies suggested that the Schiff bases are biologically active and their metal complexes showed significantly enhanced antibacterial activity against microbial strains in comparison to the free ligands, and also crystalline materials are more active than the amorphous materials. Tested compounds showed zone of inhibition ranging from 14.2 mm to 32.1 mm against the Gram-positive bacteria and between 10.1 mm and 25.7 mm against Gram-negative bacteria. The ligands (HL) show zone of inhibition ranging from 14.2 mm to 21.5 mm against Gram-positive bacteria and from 10.1 mm to 22.6 mm against Gram-negative bacteria. It has been observed that the metal complexes showed increased zone of inhibition against the bacterial strains compared to ligands. On the basis of zone of inhibition produced against the test bacterium, compound Bu_2_Sn(L^3^)_2_ was found to be the most effective against* B. cereus*,* Staphylococcus *spp.,* E. coli*, and* Klebsiella *spp.with zone of inhibition of 32.1 mm, 31.2 mm, 16.4 mm, and 18.5 mm, respectively (Table 8). This also showed that the antibacterial activity of ligands is greatly enhanced when it is coordinated with metal ions. Although it is difficult to make out an exact structure-activity relationship between the antimicrobial activity and the structure of these complexes, it can possibly be concluded that the chelation as well as the addition of a substrate enhances the activity of the complexes. The increase in the activity of the complexes compared to that of the ligands can be explained on the basis of Overton's concept [51] and Tweedy's chelation theory [52]. Chelation considerably reduces the polarity of the metal ion because of partial sharing of its positive charge with the donor group and possible *π*-electron delocalization over the whole chelate ring. Such chelation could enhance the lipophilic character of the metal atom, which subsequently favours its permeation through the lipid layers of the cell membrane. In general, metal complexes are more active than ligands and they may serve as principal cytotoxic species. Thus, exhibiting their broad spectrum nature can be further used in pharmaceutical industry for mankind as an antimicrobial agent after testing its toxicity to human beings.

## 4. Conclusion

A new series of organotin complexes were prepared in good yields. Based on various physiochemical and structural investigations, it was concluded that the ligands act as bidentate (no donor) forming distorted octahedral complexes with Bu_2_Sn(IV) ion. Furthermore, the current study strongly demonstrates that these complexes are more effective antibacterial agents than the parent ligands. Selectivity is observed in the activities of some compounds over particular microorganisms, which is very important for the future pharmaceutical applications in order to avoid the side effects, so we can conclude that the compounds synthesized and tested look very promising.

## Figures and Tables

**Table 1:** Analytical and physical data of the organotin(IV) complexes.

C. number	Reactants		Molar ratio	Products & colour	M.P. °C (d)	Yield (%)	Elemental analysis found (calcd.)				Mol. Wt. Found (Calcd.)
	Metal	Ligands					%Sn	%C	%H	%N	
Bu_2_Sn(L^1^)_2_	Bu_2_SnO	C_17_H_14_N_2_O_3_	1 : 2	C_42_H_44_N_4_O_6_Sn Red	164	94	14.35(14.49)	61.30(61.55)	5.44(5.41)	6.72(6.84)	824.55(819.53)

Bu_2_Sn(L^2^)_2_	Bu_2_SnO	C_14_H_16_N_2_O_3_	1 : 2	C_36_H_48_N_4_O_6_Sn Red	262	79.14	15.84(15.80)	57.50(57.54)	6.38(6.44)	7.56(7.46)	742.19(751.50)

Bu_2_Sn(L^3^)_2_	Bu_2_SnO	C_10_H_8_N_2_O_3_	1 : 2	C_28_H_32_N_4_O_6_Sn Red	174	76.81	18.43(18.57)	52.45(52.61)	5.00(5.05)	8.65(8.76)	630.88(639.29)

Bu_2_Sn(L^4^)_2_	Bu_2_SnO	C_17_H_13_ClN_2_O_3_	1 : 2	C_42_H_42_Cl_2_N_4_O_6_Sn Dark brown	210	72.39	13.03(13.36)	56.84(56.78)	4.73(4.76)	6.39(6.31)	872.54(888.42)

Bu_2_Sn(L^5^)_2_	Bu_2_SnO	C_14_H_15_ClN_2_O_3_	1 : 2	C_36_H_46_Cl_2_N_4_O_6_Sn Light brown	270	68.29	14.34(14.47)	52.59(52.71)	5.60(5.65)	6.70(6.83)	831.57(820.38)

Bu_2_Sn(L^6^)_2_	Bu_2_SnO	C_10_H_7_ClN_2_O_3_	1 : 2	C_28_H_30_Cl_2_N_4_O_6_Sn Dark brown	180–184	71.72	16.84(16.76)	47.41(47.49)	4.25(4.27)	7.85(7.91)	716.67(708.18)

**Table 2:** Important IR spectral data (cm^−1^) of Schiff bases and their corresponding organotin(IV) complexes.

Compounds	*ν*(OH)*ν*	*ν*(C=N–)	*ν*(C=O)	*ν*_as_(COO)	*ν*_s_(COO)	Δ*ν*	*ν*(Sn*←*N)	*ν*(Sn–O)	*ν*(Sn–C)
L^1^H	3090–2740 br	1620 s	1728 s	—	—	—	—	—	—
Bu_2_Sn(L^1^)_2_	—	1610 s	1722 s	1590 vs	1325 s	265	540 w	430 m	622 m
L^2^H	3100–2740 br	1625 s	1730 s	—	—	—	—	—	—
Bu_2_Sn(L^2^)_2_	—	1612 s	1726 s	1605 m	1333 m	272	545 m	426 s	640 m
L^3^H	3090–2750 br	1630 s	1720 s	—	—	—	—	—	—
Bu_2_Sn(L^3^)_2_	—	1616 s	1722 s	1601 s	1332 m	268	552 m	425 m	630 m
L^4^H	3110–2750 br	1622 s	1735 s	—	—	—	—	—	—
Bu_2_Sn(L^4^)_2_	—	1605 s	1732 s	1608 s	1330 m	278	545 s	420 w	635 m
L^5^H	3080–2730 br	1630 s	1735 s	—	—	—	—	—	—
Bu_2_Sn(L^5^)_2_	—	1612 s	1734 s	1595 vs	1326 s	269	540 w	425 w	625 m
L^6^H	3109–2730 br	1622 s	1725 s	—	—	—	—	—	—
Bu_2_Sn(L^6^)_2_	—	1610 s	1728 s	1610 vs	1340 vs	270	544 w	432 m	632 m

br: broad, vs: very sharp, v: sharp, m: medium, and w: weak.

**Table 3:** ^
1^H NMR spectral data^a^ of the ligands and their corresponding organotin(IV) complexes.

Compounds	Chemical Shift (*δ*, ppm)
L^1^H	11.50 (s, 1H, COOH), 4.25 (t, 1H, N–CH–CH_2_–), 3.08 (d, 2H, –CH_2_–Ph), 8.01 (s, 1H, NH), 7.20–7.80 (m, 9H, aromatic).

Bu_2_Sn(L^1^)_2_	4.21 (s, 1H, N–CH–CH_2_), 3.10 (d, 2H, –CH–CH_2_–Ph), 8.03 (s, 1H, NH), 7.10–7.75 (m, 9H, aromatic); 1.60–1.15 (br m. 6H, –CH_2_–CH_2_–CH_2_–); 0.82 (br t, 3H, CH_3_ of butyl groups).

L^2^H	11.39 (s, 1H, COOH), 3.96 (d, 1H, N–CH–CH–), 2.10–2.16 (m, 1H, CH_3_–CH–CH_2_–), 1.50–1.60 (m, 2H, –CH–CH_2_–CH_3_), 8.05 (s, 1H, NH), 0.85 (d, 3H, –CH–CH_3_), 0.95 (t, 3H, –CH_2_–CH_3_), 7.25–7.82 (m, 4H, aromatic).

Bu_2_Sn(L^2^)_2_	3.98 (d, 1H, N–CH–CH), 2.08–2.16 (m, 1H, CH_2_–CH–CH_3_), 0.80 (d, 3H, –CH–CH_3_), 1.48–157 (m, 2H, CH–CH_2_–CH_3_), 0.99 (t, 3H, –CH_2_–CH_3_); 8.10 (s, 1H, NH), 7.22–7.80 (m, 4H, aromatic); 1.65–1.21 (br m. 6H, –CH_2_–CH_2_–CH_2_–); 0.80 (br t, 3H, CH_3_ of butyl groups).

L^3^H	11.28 (s, 1H, COOH), 4.30 (s, 2H, N–CH_2_–), 8.02 (s, 1H, NH), 7.28–7.80 (m, 4H, aromatic).

Bu_2_Sn(L^3^)_2_	4.38 (s, 2H, N–CH_2_–), 8.05 (s, 1H, NH), 7.25–7.78 (m, 4H, aromatic); 1.62–1.18 (br m. 6H, –CH_2_–CH_2_–CH_2_–); 0.84 (br t, 3H, CH_3_ of butyl groups).

L^4^H	11.42 (s, 1H, COOH), 3.97 (d, 1H, N–CH–CH_2_–), 3.15 (d, 2H, –CH_2_–Ph), 8.08 (s, 1H, NH), 7.20–7.85 (m, 8H, aromatic);

Bu_2_Sn(L^4^)_2_	4.02 (t, 1H, N–CH–CH_2_), 3.08 (d, 2H, –CH_2_–Ph), 8.10 (s, 1H, –NH–), 7.20–7.75 (m, 8H, aromatic); 1.60–1.15 (br m. 6H, –CH_2_–CH_2_–CH_2_–); 0.80 (br t, 3H, CH_3_ of butyl groups).

L^5^H	11.42 (s, 1H, COOH), 3.99 (d, 1H, N–CH–CH), 2.08–2.14 (m, 1H, CH_3_–CH–CH_2_), 1.42–1.58 (m, 2H, CH–CH_2_–CH_3_), 8.10 (s, 1H, NH), 0.82 (d, 3H, –CH–CH_3_), 0.98 (t, 3H, –CH_2_–CH_3_), 7.20–7.80 (m, 3H, aromatic).

Bu_2_Sn(L^5^)_2_	4.01 (d, 1H, N–CH–CH), 2.10–2.20 (m, 1H, CH_3_–CH–CH_2_), 0.80 (d, 3H, –CH–CH_3_), 1.45–1.58 (m, 2H, –CH–CH_2_–CH_3_), 1.02 (t, 3H, CH_2_–CH_3_), 8.05 (s, 1H, NH), 7.20–7.82 (m, 3H, aromatic); 1.64–1.18 (br m. 6H, –CH_2_–CH_2_–CH_2_–); 0.82 (br t, 3H, CH_3_ of butyl groups).

L^6^H	11.35 (s, 1H, COOH), 4.32 (s, 2H, –CH_2_–), 8.12 (s, 1H, NH), 7.20–7.76 (m, 3H, aromatic).

Bu_2_Sn(L^6^)_2_	4.40 (s, 2H, N–CH_2_–), 8.10 (s, 1, NH), 7.28–7.76 (m, 3H, aromatic); 1.65–1.21 (br m. 6H, –CH_2_–CH_2_–CH_2_–); 0.85 (br t, 3H, CH_3_ of butyl groups).

^a^Chemical shift (*δ*) in ppm: Multiplicity is given as: s = singlet, d = doublet, t = triplet, q = quartet, m = complex pattern, br = broad.

**Table 4:** ^
13^C NMR spectral data of the ligands and their corresponding organotin(IV) complexes.

Compounds	Chemical shift in (*δ* ppm)					
	COOH	CH	C=N	CH_2_/CH_3_	Aromatic carbons	Sn–^*α*^CH_2_–^*β*^CH_2_–^*γ*^CH_2_–^*δ*^CH_3_
L^1^H	176.8	67.8	163.4	38.2	149.2, 135.8, 133.0, 128.6, 127.5, 126.3, 124.6, 122.9, 120.1	—

Bu_2_Sn(L^1^)_2_	185.4	65.7	155.2	39.4	150.0, 136.1, 132.6, 128.2, 127.1, 126.5, 124.4, 123.3, 120.2	C-*α*, 22.2; C-*β*, 25.6; C-*γ*, 22.4; C-*δ*, 13.4

L^2^H	178.5	65.7	162.4	16.3, 21.3	145.8, 133.2, 130.5, 126.2, 124.0, 119.3	—

Bu_2_Sn(L^2^)_2_	184.9	66.2	154.6	17.4, 22.1	146.2, 132.9, 130.8, 126.54, 124.3, 120.1	C-*α*, 21.4; C-*β*, 25.3; C-*γ*, 21.6; C-*δ*, 13.5

L^3^H	176.1	—	163.6	52.4	148.5, 131.0, 129.8, 125.3, 122.9, 119.1	—

Bu_2_Sn(L^3^)_2_	183.7	—	154.5	58.3	149.0, 132.5, 129.6, 125.7, 123.4, 119.6	C-*α*, 22.1; C-*β*, 25.2; C-*γ*, 22.3; C-*δ*, 14.1

L^4^H	178.5	65.7	162.4	39.1	150.2, 136.1, 132.8, 129.5, 127.3, 126.4, 123.2, 122.4, 119.8	—

Bu_2_Sn(L^4^)_2_	184.5	66.2	154.5	38.7	149.5, 135.7, 132.1, 130.4, 126.9, 126.2, 123.5, 122.1, 120.3	C-*α*, 22.4; C-*β*, 26.7; C-*γ*, 22.8; C-*δ*, 14.6

L^5^H	177.5	66.2	163.1	17.3, 20.5	147.7, 132.8, 131.3, 126.6, 123.8, 120.3	—

Bu_2_Sn(L^5^)_2_	184.1	67.4	153.8	18.0, 20.2	148.1, 132.4, 129.7, 126.5, 124.0, 120.2	C-*α*, 22.0; C-*β*, 26.1; C-*γ*, 22.5; C-*δ*, 13.4

L^6^H	176.9	—	162.9	55.4	149.5, 132.8, 128.9, 125.7, 123.0, 120.3	—

Bu_2_Sn(L^6^)_2_	182.8	—	155.4	56.1	148.2, 133.1, 128.5, 125.8, 122.7, 120.5	C-*α*, 21.2; C-*β*, 25.3; C-*γ*, 22.24; C-*δ*, 13.7

**Table 5:** X-ray powder diffraction data for compounds (L^1^H) and (L^2^H).

S. number	2*θ*	“*d*” (Å)	Intensity (count)	FWHM	*D* ^*^ (nm)
L^1^H					
1	13.517	6.54547	385.6	0.5425	25.381
2	14.936	5.92655	482.3	0.4308	31.912
3	16.873	5.25031	216.5	0.7307	18.770
4	21.492	4.13128	323.9	0.7307	18.643
5	22.046	4.02874	453.9	0.6139	22.169
6	22.521	3.94474	289.4	0.6139	22.151
7	24.025	3.70113	301.8	0.6139	22.091
8	24.805	3.58649	162.8	0.6139	22.059
9	25.067	3.54964	205.2	0.6139	22.047
10	26.359	3.37851	150.7	0.6139	21.991
11	27.324	3.26131	228.2	0.486	27.722
12	28.440	3.13581	217.5	0.7166	18.756
13	32.024	2.79258	112.5	0.7166	18.598

L^2^H					
1	11.495	7.69156	128	0.3461	39.860
2	13.137	6.73368	236.3	0.3806	36.191
3	14.870	5.95279	807.9	0.2844	48.343
4	16.683	5.30969	228.6	0.3597	38.139
5	21.263	4.17518	222.4	0.3597	37.885
6	22.035	4.03068	286.1	0.5986	22.736
7	23.706	3.75021	263.9	0.5544	24.476
8	24.902	3.57273	521.9	0.3499	38.695
9	26.232	3.39454	156.1	0.3499	38.593
10	27.114	3.28608	717.3	0.2874	46.899
11	31.637	2.82587	144.2	0.4665	28.597
12	37.570	2.3921	84.3	0.6166	21.289

^*^ *D*: crystallite size (in Å) and *d*: lattice spacing.

**Table 6:** Important bond lengths of compound [Bu_2_Sn(L^2^)_2_].

S. number	Atoms	Actual (Å)	Optimal (Å)
1	Sn(1)–C(44)	2.2339	2.1620
2	Sn(1)–C(47)	2.2064	2.1620
3	N(9)–Sn(1)	2.1050	—
4	N(5)–Sn(1)	2.1010	—
5	Sn(1)–C(44)	2.2339	2.1620
6	Sn(1)–O(6)	2.0644	—
7	O(6)–C(7)	1.3666	1.3380
8	Sn(1)–O(2)	2.0581	—
9	O(2)–C(3)	1.3639	1.3380
10	Sn(1)–C(47)	2.2064	2.1620
11	N(5)–C(14)	2.1680	1.2600
12	C(4)–N(5)	1.4923	1.4700

**Table 7:** Important bond angles of compound [Bu_2_Sn(L^2^)_2_].

S. number	Atoms	Actual (°)
1	C(47)–Sn(1)–C(44)	117.1540
2	C(47)–Sn(1)–N(5)	85.7227
3	C(47)–Sn(1)–N(9)	124.3910
4	C(47)–Sn(1)–O(6)	68.5077
5	C(47)–Sn(1)–O(2)	141.0197
6	C(44)–Sn(1)–N(5)	80.4837
7	C(44)–Sn(1)–N(9)	78.7094
8	C(44)–Sn(1)–O(6)	138.6752
9	C(44)–Sn(1)–O(2)	88.6745
10	N(5)–Sn(1)–N(9)	148.9524
11	N(5)–Sn(1)–O(6)	139.5356
12	N(5)–Sn(1)–O(2)	69.4566
13	N(9)–Sn(1)–O(6)	67.5865
14	N(9)–Sn(1)–O(2)	87.2268
15	O(6)–Sn(1)–O(2)	112.0005

**Table 8:** Antibacterial activity of ligands and their organotin(IV) complexes.

Schiff bases/complexes	Zone of inhibition (mm)							
	Gram-negative				Gram-positive			
	*E. coli *		*Klebsiella *spp.		*B. cereus *		*Staphylococcus *spp.	
	500 ppm	200 ppm	500 ppm	200 ppm	500 ppm	200 ppm	500 ppm	200 ppm
L^2^H	18 ± 0.6	15 ± 0.1	22 ± 0.6	17 ± 0.1	14 ± 0.2	12 ± 0.9	23 ± 0.2	20 ± 0.7
Bu_2_Sn(L^2^)_2_	25 ± 0.7	22 ± 0.4	30 ± 0.3	26 ± 0.8	20 ± 0.7	18 ± 0.4	30 ± 0.1	25 ± 0.5
L^3^H	12 ± 0.1	10 ± 0.3	15 ± 0.1	13 ± 0.6	20 ± 0.1	18 ± 0.6	21 ± 0.5	18 ± 0.6
Bu_2_Sn(L^3^)_2_	16 ± 0.4	15 ± 0.6	18 ± 0.5	16 ± 0.4	32 ± 0.1	28 ± 0.3	30 ± 0.2	27 ± 0.2
L^5^H	14 ± 0.3	12 ± 0.9	15 ± 0.2	12 ± 0.1	24 ± 0.6	20 ± 0.1	19 ± 0.1	17 ± 0.1
Bu_2_Sn(L^5^)_2_	20 ± 0.5	18 ± 0.1	20 ± 0.5	18 ± 0.5	28 ± 0.3	23 ± 0.4	29 ± 0.4	25 ± 0.6
L^6^H	11 ± 0.3	13 ± 0.2	10 ± 0.1	8 ± 0.5	20 ± 0.5	17 ± 0.8	18 ± 0.6	15 ± 0.2
Bu_2_Sn(L^6^)_2_	16 ± 0.2	15 ± 0.5	11 ± 0.8	10 ± 0.9	24 ± 0.9	19 ± 0.5	20 ± 0.1	18 ± 0.4
Streptomycin	28 ± 0.1	23 ± 0.3	26 ± 0.3	20 ± 0.5	26 ± 0.8	22 ± 0.1	32 ± 0.1	26 ± 0.3
DMSO	0	0	0	0	0	0	0	0
